# Maximizing Student Clinical Communication Skills in Dental Education—A Narrative Review

**DOI:** 10.3390/dj10040057

**Published:** 2022-04-01

**Authors:** Rod Moore

**Affiliations:** Institute of Dentistry and Oral Health, Aarhus University, 8000 Aarhus C, Denmark; rod.moore@dent.au.dk; Tel.: +45-22-35-78-95

**Keywords:** active listening, communication skills, dental education, empathy, health consultations, longitudinal learning, motivational interviewing, patient-centered communication, role-play feedback, video feedback

## Abstract

Dental student training in clinical communication skills and behavioral aspects of treatment are lauded as clinically meaningful in the dental education literature. However, many dental school curricula still only provide didactic, one-time coursework with multiple choice examination assessment and little or no student skill-activating activities. This article aims to review literature relevant to optimizing clinical communication and behavioral skills in dental education. The review summarizes findings of several relevant reviews and usable models to focus on four themes: (1) special characteristics of dentistry relevant to communication skill needs, (2) essential components of dental student learning of communications skills, (3) clinical consultation guides or styles and (4) optimal curricular structure for communication learning effectiveness. Contexts of communications in the dental chair differ from medical and other allied health professions, given the current mostly dentist-dominant and patient-passive relationships. Patient-centered communication should be trained. Dental students need more practical learning in active listening and patient-centered skills including using role-play, videotaping and ultimately, real patient training. Medical consultation guides are often unwieldy and impractical in many dental contexts, so a shortened guide is proposed. Communication skills need to be learned and taught with the same rigor as other core dental skills over the entire course of the dental curriculum.

## 1. Introduction

Effective communication skills, that is, accurate listening or observation and focused verbal or non-verbal response, enhances efficiency of diagnoses, ethical clinical decision making, positive clinical outcomes, promotes patient use of services, and patient–clinician satisfaction [1,2,3]. It also helps to decrease patient anxiety and pain perception [4]. Conversely, poor communication is the most common reason for dissatisfaction with care, promotion of distrust, including malpractice [5], and is the most common cause for termination of the relationship [6]. 

Most of the literature about student learning of clinical communication skills appears in allied health, especially for medical and nursing students. This literature is vast compared with similar dental educational literature [7,8]. The role of the dentist or dental student and the context of interactions with patients reveals some very special conditions, such as supine dental chair position and that the patient’s mouth is often occupied. These among others, make this role unique among health care workers. Moreover, unlike modern medical care situations, the patient role is most often passive and the relationship is dentist dominated [9,10]. For this reason, it is important not to extrapolate allied health findings and draw conclusions for dental student communication skills learning. Furthermore, given this persistent dentist-dominated relationship over time, many educators emphasize the need for dental students to increase practical learning in active listening and patient-centered skills [8,9,10,11]. Patient-centered communication has shown several advantages in the medical literature, including improved patient recall, compliance and satisfaction [11]. Research on advantages of patient-centered communication in dentistry is scant in comparison, but includes decreased patient dental anxiety [4,12], decreased perceived operative pain [4] as well as improved oral hygiene and periodontal compliance [13,14].

Finally, dental communication skills have traditionally been taught as one time didactic coursework, with little or no practical components prior to 2010 [7]. Although this has changed considerably, some dental education experts [7,15,16] have called for more longitudinal teaching that would follow students’ clinical development through the course of the dental curriculum.

The following review attempts to synthesize knowledge gained from the literature specifically related to dental student learning of clinical communication skills in order to make recommendations towards optimization. Knowledge synthesis is important in health care research and practice because it can help contextualize and make sense of evidence that might not be obvious for some, yet can be influential for, as in this case, educational policy and practice. This review placed relatively few restrictions on inclusion criteria in order to sample essential or underemphasized points in the literature that speak to the unique nature and expected quality of teaching dental clinical communication skills. As described in the paragraph above, several reviews about dental school communication skills training, among others referred to below, seemed to share several conceptual agreements over the time between their respective publications. These temporal reiterations appeared important to their respective authors, but sometimes lacked depth of description contextualized to the daily needs of dental students. Four themes appeared to provide important contexts for optimizing learning of communication skills of dental students. The following review research agenda and search aimed to improve understanding of (1) special characteristics of dentistry relevant to communication skills needs, (2) essential components of dental student learning of communications skills, (3) clinical consultation guides or styles used in communication skills learning and (4) optimal curricular structure for clinical communication learning effectiveness. The hope of this review is to bring into focus these important themes of dental communications skills training that could improve educational policy and practice for optimizing dental student learning in the clinic. 

## 2. Materials and Methods

In order to make a list of considerations that would be helpful for optimizing communication skills training (CST) in dental education, the review especially focused on including (1) review studies, (2) 2010 or later and (3) English language articles that examined CST components in dental education. The initial search query was run in PubMed. Text terms used in the blanket query were “communication skills +/in dental education”. The search retrieved 369 articles. A Cochrane Library search contributed 7 more articles after duplicates were culled out. Since most of the review articles in that batch were before or included 2019, Google Scholar was used to repeat the same blanket query search for updates from 2020 to November 2021 to supplement previous searches. Google Scholar also has another convenient feature with its selection choice for “reviews”, which was helpful to refine the overall search for the most up-to-date review articles. The Google Scholar search found 10 more potentially relevant articles after duplicates were culled. Thirteen articles were identified as bona fide review articles related to learning or teaching of communication skills in dental education. The reason review articles were the focus of selection from the initial search was in order to explore themes from an overview perspective and in context, rather than search out of context for “uniqueness of dental communications”, “essential components of dental communication skills training”, “clinical consultation guides in communication skills training” or “optimal dental curricular structure for communication skills training”. Thus, presently selected review articles and their references were used to sample the literature that contained contextual information about any of the four themes. Seven of the studies were systematic reviews, one was a systematic scoping review and five were narrative reviews. 

Since review articles would report on content of CST components at the time of their publication, all articles before 2010 were considered outdated, which eliminated one of the systematic reviews [17]. The remaining twelve bona fide reviews were used to synthesize the thematic analysis. Dental literature references from these reviews also accounted for 519 potential references from which to draw detailed information. In this qualitative research review approach, an attempt was made both to maintain the frame of reference of the themes as well as to provide a critical exploration/discovery of the depth of the themes.

## 3. Results

Findings in the present review are summarized and tabled in Table 1 according to literature relevant to the four chosen themes. The four themes are described in a detailed narrative in the Discussion section as well.

## 4. Discussion

### 4.1. Special Characteristics about the Dental Clinic and Communication

Sociolinguists Coleman and Burton [18] originally pointed out that there is considerable difference between dental and medical consultations in that the latter are hands-on treatment sessions only in exceptional cases. This has also been echoed recently in the dental literature [13,19,20]. The functions of the physician in a typical consultation are to investigate symptoms and patient perceptions, aid diagnosis, prescribe treatment, review progress and give information and advice. The structure as described by sociolinguist Long and others [20,21] is a sequence of phases, which closely resemble other decision-making schemes in other disciplines: (1) greeting the patient; (2) discovering the patient’s reasons for attending; (3) conducting a physical or verbal examination (or both); (4) sharing information with the patient; (5) detailing further courses of action (i.e., prescription of drugs or referral); and (6) closing the consultation. More recently, it has evolved towards patient-centered communication and is called the “six-functions model” as reviewed by King and Hoppe [11]: (1) fostering the relationship, (2) gathering information, (3) providing information, (4) making decisions, (5) responding to emotions, and (6) enabling disease- and treatment-related behavior in closure. The emphasis upon one phase or another varies from case to case, often requiring more work on building rapport in a relationship throughout the consultation. An initial consultation with the dentist also has all of these functions. 

On the other hand, one crucial difference is that there is actual administration of hands-on dental treatment in nearly all phases of dentist–patient interaction, including initial consultations [13,18,19,20]. Patient-centered interactions requiring response to emotions force dentists to also take into account patients’ perceived feelings of need for control during treatment in the dental chair, among other things [20]. Thus, in a dental private practice setting, only initial contact with a new patient for the first time truly resembles a medical consultation, unless there are special circumstances for follow-up consultations due to anxiety or special care needs. In a 2010 systematic review of dental communication skills training, Carey et al. [7] pointed out the necessity for communications skills learning to be extended to include intra-operative communication, rather than solely based on learning skills for initial consultations with patients. Since most communication occurs during actual treatment phases, rather than in initial consultations, it seems logical that dental students should be prepared for both. 

Furthermore, all dental patients are recalled for periodical check-ups and preventive treatments, unlike most medical patients. This also affects the context of dentist–patient communication compared with the context of medical consultations. In a 2019 major systematic review of communication coursework in dental education, Khalifah and Celenza [8] also point out the necessity for communication skills to be extended to include intra-operative communication, concurring with Carey et al.’s findings. They also go on to study dental communication skill categories and other essential components of dental student learning.

### 4.2. Essential Components of Dental Student Learning of Communication Skills

**Figure 1:**
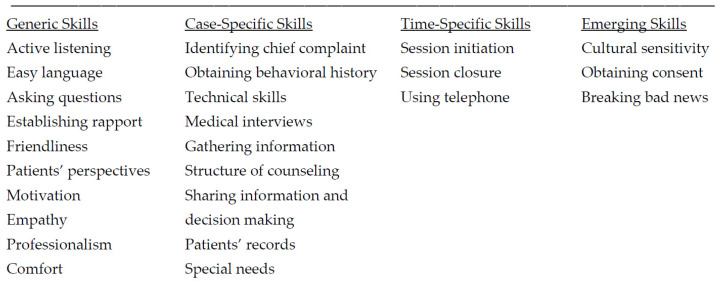
Twenty-six communication skills in four categories adapted from Khalifah and Celenza [8]. Generic skills are those to be used at any dental visit and must become natural habits of the dentist. Case-specific skills regard individual cases and situations and vary according to patient and case. Time-specific skills are appropriate at certain times in a consultation. Emerging skills are skills to be applied in distinctive cases with special considerations.

Khalifah and Celenza [8] identified 26 communication skills that fell under four categories as listed below (see Figure 1 for details).

Khalifah and Celenza also tabled each of the 50 relevant studies for type of communication skills taught, teaching method (e.g., role-play, video supervision, lectures) and assessment method and outcomes [8]. From these, we obtain a picture of the specific contexts for learning communication skills in dentistry and being able to rate them according to quality. In common with most of the articles reviewed and tabled by Khalifah and Celenza, “active listening”, gathering information, establishing rapport, empathy, professionalism and motivation were predominant [8]. According to Khalifah and Celenza [8], the highest quality studies put the patient’s perspective at the center of communications, no matter how short or long the duration of consultation or needs within the context described. 

#### Patient-Centered Care—Active Listening and Empathy

People tend to overestimate their listening comprehension, suggesting that they may not perceive listening as a skill requiring development in the same way that speaking, reading, writing or manual techniques are skills acquired through instruction, effort and time [22]. Active listening is the major skill promoted in patient-centered communication [8,23,24,25,26,27]. Active listening was initially promoted among others, by psychologist Carl Rogers [27,28]. Rogers argued that active listening was the most effective way to explore and understand a patient’s problems and help them as well. A normal first reaction of most people in thinking about listening as a possible therapy for dealing with human problems is that listening is thought of as passive and insufficient. They think that listening does not communicate anything to the speaker. However, by consistently listening and verifying what one hears with a speaker, the listener is conveying the idea: “I’m interested in you and I think that what you have to say is important. I respect your thoughts, and (even if I may not agree with them), I know that they are valid for you. I’m not trying to change you or evaluate you. I just want to understand you.” Active listening promotes empathy with the speaker, which promotes positive outcomes [11,24]. Active listening involves learning to work with both non-verbal as well as verbal communication in order to “mirror” for a patient, that is, to verify meaning by summarizing and reformulating statements for clearer mutual understanding. Active listening is a “healthy combination” of critical listening, reflective listening and passive listening. Active listeners are critical in trying to interpret a message and evaluate the speaker’s emotions and non-verbal cues; reflective listening helps the speaker to “feel heard”; and silence and pauses in passive listening signals to the speaker that there is uninterrupted time for them to communicate their message [29]. Active listening is used both in initial consultations and during communications in active dental treatment. Adapting active listening as a central element in a communication skills curriculum is not only essential to optimizing one-on-one communication between the student dentist and patients, but it also signals a philosophical change from doctor-centered to patient-centered consultations and treatment [11,15].

Patient-centered care (PCC) has grown out of observations that active listening and empathy maximize clinical communication and health outcomes and was first officially espoused by the Institute of Medicine in 2001 [11]. King and Hoppe’s 2013 extensive review of patient-centered care indicated a consensus about what constitutes ‘‘best practice’’ for communication in clinical encounters, the so-called “six-functions model” described above, which basically pervades all consultation models and styles that will be described below. King and Hoppe [11] surmised that there was abundant evidence in the medical literature supporting the importance of patient-centered communication skills as a dimension of physician competence. They cited evidence of positive outcomes about patient recall, understanding, satisfaction and adherence to therapy [11]. King and Hoppe stated that “efforts to enhance teaching of communication skills to medical trainees would likely require significant changes in instruction at undergraduate and graduate levels, as well as changes in assessing the developing communication skills of physicians.” An added critical dimension is faculty understanding of the importance of communication skills, and their commitment to helping trainees develop those skills [11].

In a systematic review of PCC in dentistry, Scambler et al., [10] concluded it is about delivering humane care involving good communication and shared decision-making. However, they noted there was no evidence in the dental literature suggesting that the concept is either clearly understood or empirically and systematically assessed in dental settings. They presented a model of four levels of information and choice provision and/or agreement between dentist and patient. Level 1 is one-way information from the dentist. Level 2 is when patient makes informed choice among informed treatment options. Level 3 is when patients are given tools to make the choice themselves, under advisement. Finally, Level 4 is when the patient is in full control of their care and capable of making informed choices they wish or do not wish to achieve. 

The model does not assume that all patients would want, or be happy with a Level 4 approach, that is, may not be their hierarchical endpoint.

Another systematic review by Mills et al. [9] revealed a lack of understanding of PCC in dentistry, and in particular, general dental practice. Mills et al. [10] pointed to a poor evidence base and no support for the use of then current patient reported outcome measures as indicators of patient-centeredness. However, Mills et al. did find that special dentistry qualitative research about treatment of phobic and economically disadvantaged patients provided some evidence of good outcomes using patient-centered communication for these vulnerable populations.

In summary, unlike the medical literature, the dental literature on patient-centered care and communication is scant. This possibly reflects values of dental education and of the profession as a whole in the reluctance to adopt what has proven to be in the medical profession and in society in general, a developmental paradigm shift. 

### 4.3. Other Essential Aspects of Education of Dental Students in Communication Skills

#### 4.3.1. Role-Play vs. Clinical Video Projects

In their seminal review of teaching dental students communication skills, Khalifah and Celenza expressed disappointment that CST was still being taught using passive lecture techniques and that assessment was by written examination [8]. It is generally agreed that these teaching formats limit student learning and create difficulties in assessing practical communication skills [7,8,30]. Khalifah and Celenza [8] found that role-playing, patient interviewing and clinical observation were used in CST, especially in the clinical years of dental study. Two of the studies they cited found these methods were useful in distinguishing effective from ineffective communication skills, and dental students showed positive attitudes toward actively learning skills, regardless of using role-play or clinical video supervision [31,32]. 

However, the Carey et al., review of dental communication skills training [7] indicated that it was best that skills be evaluated during interactions with real patients, thus calling for at least some clinical coursework after initial role-play in earlier coursework. Contrary to scripted role-play, the nuances of how patient interactions live may provide a more realistic sense of clinical situations, and thus is naturally more motivating for student learning [7]. Khalifah and Celenza also assessed that video supervision of actual patient–dentist interactions and especially in one to three concentrated course days, were best for learning optimal communication skills [8]. Currently, there are several video platforms that can be used for secure storage of video clips obtained during the students’ interactions with patients, given proper written consent. These are also excellent resources for case presentations in plenum, in order for teachers and students to discuss many clinical situations filmed within the learning group.

#### 4.3.2. The Role of Clinical Instructors in Communication Skills Learning

Burkert’s review [33] reported that the ultimate learning of optimal clinical communication skills requires teachers to be “good role models, effective supervisors, powerful tutors and supportive persons who use dynamic and diverse teaching methods and have an individual approach to educating their students.” Ayn et al. [15] also underscored the role of the clinical instructor in modeling clinical communication skills as a form of learning in which there is very little educational institution control over its quality. If students are taught by instructors with poor communications skills, there is a greater chance that the student will learn less optimal communication skills. Ayne et al. [15] described it as “Investing in faculty as well as student communication skill development provides an opportunity for positive role-modeling and patient-focused rapport between students and instructors.” A major takeaway from the review was that instructors, as well as students, need to value good communication skills and be willing to adapt to patient-centered communication.

### 4.4. Clinical Consultation Guides and Styles Used in Communication Skills Learning

The literature on structural checklists or guides for health care personnel for making consultations is in agreement with a central premise to facilitate patient-centered communication. However, from the start, there is some controversy, since the overwhelming amount of literature about structural guides for clinical student learning is mainly in health science education literature other than dentistry. There is not nearly this amount of literature for dental consultation formats and styles. The descriptions in the first theme at the beginning of this review regarding special characteristics of the dental clinical environment and special clinical communication conditions fundamentally refers to a difference between medical and dental consultations and that important emphasis is also needed on intra-operative communication in dentistry. The intention with the descriptions below is to provide dental students with a helpful, relevant guide in learning the case wise temporal structure of longer patient consultations. Hopefully, the following narrative will provide some needed synthesis about this theme and can clarify directions for teaching dental students these skills. 

According to the literature, there are two main types of models of patient-centered consultation strategies that have been developed as guidelines for interviewing patients for diagnostics and/or health promotion: motivational interviewing (MI) [34,35,36] and the Calgary–Cambridge Guide (C-CG) [37,38] or similar scales such as the Macy Foundation or Manitoba models [8,13,19]. All consultation guides or styles require information gathering, active listening, empathy and relation building as a premise fundamentally similar to the “six functions” as described above [11]. They provide structure to learning how to conduct systematic interviews mostly in relation to anamnestic consultations, in health promotion or as needed in specific cases of clinical communication about behavioral or medical problems. They differentiate specific communication skills (e.g., being attentive, using appropriate language, tending to patients’ comfort), while also incorporating, to a lesser or greater degree, assessment of communication skills and particularly students’ self-assessment, since they tend to be checklists.

#### 4.4.1. Motivational Interviewing Consultation Style

Motivational interviewing (MI) was perhaps the earliest consultation model strategy. It came about from a desire of health professionals to support patients to achieve desired behavior change in special needs (e.g., decreased smoking, increased oral hygiene) [14,34,39]. MI is defined as ‘‘a person-centered counseling style for addressing the common problem of ambivalence about change.’’ [36] MI originally arose from efforts of Miller and colleagues to help patients with risky alcohol behavior [35,36]. The interview structure assumes initiation of rapport building, discussion of a patient’s motivations and information sharing and use of Roger’s humanistic psychology techniques [34]. Miller and others [34,36] described the strategy as identifiable by the acronym “OARS”, i.e., asking Open-ended questions, providing Affirmations (positive feedback), use of Reflective listening, and discussion of Summaries. These are described in detail by Miller and others [34,36] as the following.

- Open-ended questioning—In contrast to closed questions, which generally require a simple yes/no or numeric answer, open questions do not direct a patient to respond in a particular manner. Instead, they enable a patient to think through and provide richer, fuller responses. The conversation should be started with words such as how or what or describe so that the patient does most of the talking.
- Affirmations—Sincere affirmations can help build a stronger relationship with a patient. These are the statements and gestures of health care workers that help the patient to recognize strength and acknowledge behaviors that lead to positive change, regardless of how big or small.
- Reflective listening—Akin to active listening, this demonstrates that the clinician has accurately heard and understood a patient’s communication. This relational empathy encourages further exploration of problems and feelings and encourages “change talk”.
- Summaries reinforce what has been said. Summary statements include trying to obtain the full picture of a patient’s behavior and checking with the patient to make sure that they feel the health care professional has reflected their situation accurately. Summarizing helps in integrating the communication that has occurred between the patient and provider.

#### 4.4.2. Macy Model of Doctor–Patient Communication

The Macy Foundation model identifies seven parts of the medical encounter (preparing, opening, gathering information, eliciting patient perspectives, educating patients, agreeing on treatment plans and closing the interview) and focuses on relationship-building skills and interview-managing skills [8]. The Manitoba model is conceptually and structurally similar to the Macy model [8].

#### 4.4.3. Calgary–Cambridge Guide (C-CG) to Health Consultations

**Figure 2:**
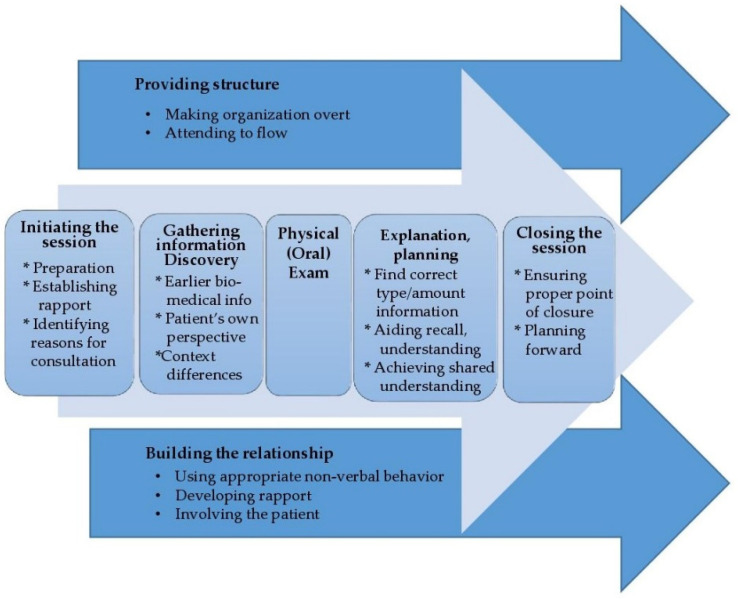
Calgary–Cambridge Guide Domains (adapted from Silverman et al. [35] and Kurtz et al. [34].

The Calgary–Cambridge Guide [37] has often been used for training mostly medical and nursing students in the essential steps for communication in medical consultation with patients. It is used in some dental educational programs as well. Unlike MI, the C–CG is not necessarily seen as a tool for promoting motivation and behavioral change in patients’ health behaviors. It is mostly seen as a checklist approach to assure maximum patient contact and useable information in making diagnoses and treatment decisions. Depending on the version, the C–CG has been a 59 to 71 item checklist that can be scored by an observer to assess student behavior in a consultation on a 0–2 Likert scale [40]. The C–CG has been described as unwieldy given the number of items [40]. Several attempts have been made to economize it. A potentially useful and significantly shortened measure was recently adapted for learning/assessing clinical consultation skills in students at a multiple health care provider setting using a 12-item observation scheme (Observation Scheme 12, OS-12) [40]. OS-12 adjusts the Cambridge–Calgary Guide to 12 action parameters assessed on a 0–4 scale (48 max.) and if more detail is needed, provides detail of micro-skills. The items in combination or singularly are more concise than the C–CG, but correspond to C–CG domains (Figure 2) as shown in Table 2 below.

**Figure 3:**
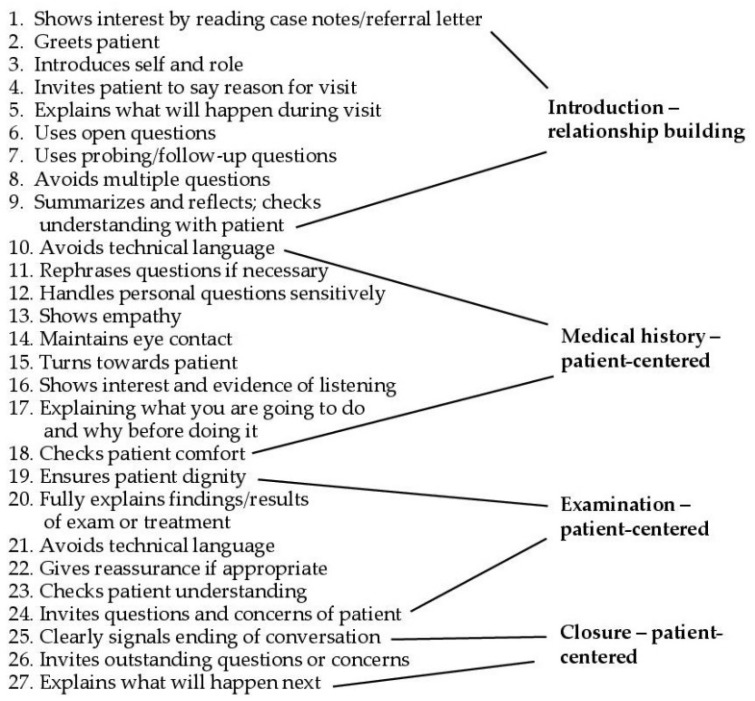
Dental Consultation Communications Checklist (DCCC) from Sangappa, 2013.

There is also a dental version inspired by the C–CG, the Dental Consultation Communications Checklist (DCCC) [41,42,43]. The original version was formulated and tested on 43 third year English dental students by Theaker et al. in 2000 [43]. It was then validated on 204 Iranian clinical dental students in 2015 [41]. This original version was 31 items, where the patient was also asked for an assessment in three of the items. A version of DCCC was tested in 2013 [42] and validated a 27 item version used for assessment of (see Figure 3) an experimental intervention group versus a control group of Indian dental students. Sangappa et al. [44] more recently created another 40-item version that was modified and factor analyzed as both an interview guide and an assessment tool. The authors of these studies suggest that DCCC is appropriate for use in guiding consultations, feasible for routine use as assessment tools for dental students and reliable [41,43,44]. Although not as complicated or lengthy as the C–CG, even the DCCC might be unnecessarily tedious for routine dental student use and seems unwieldy in intra-operative consultations.

#### 4.4.4. The “Four Habits” Model Plus One Extra Good Dentist Habit 

Torper et al. [20] present a consultation model for dentistry that they propose is “applicable for most types of visits, patients and problems”. The original medical model of the Four Habits model (4H) as described by Frankel and Stein [45] was modified by Torper et al. [20] to the specific structure and content of dental visits. The 4H model proposes four phases: (1) invest in the beginning (relationship building), (2) elicit the patient’s perspective, (3) demonstrate empathy and (4) invest in the end (successful closure). It is thus generally similar to C–CG. Facilitate Perceived Control (FPC) was added to the model by Torper et al. due to its crucial importance in dental visits, so Torper et al. named the model the “Four-plus-one Habit model for Dental Visits (4 + 1HD)” [20]. The model was intended to become a flexible framework for communication skills training at all levels of dental education, given its simplicity [20]. Torper et al. called for more research to validate and test the model in various clinical and dental educational settings. Although the first four habits are largely comparable to the Dental Consultation Communications Checklist, Torper et al.’s FPC habit does recognize the need for specific patient communication in situations where the patient might have a tendency to perceive powerlessness or loss of control over a situation, such as with fear of anesthetic injections, drilling or other treatment procedures [13]. Thus, Torper et al.’s 4 + 1HD attempted to combine the best of the Four Habits model [20], the “six functions” [11] and the Cambridge–Calgary Guide and weld them into a model of pre-clinical consultation and intra-operative communication. However, even though this model better addresses the context of dental clinical consultations, it still has a checklist of up to 27 habit components and seems nearly as tedious as the DCCC.

Given the need described above to find a concise yet flexible guide for training dental student consultations, the following suggestion is made, given special communication needs in the dental clinic. In Khalifah and Celenza’s systematic review, they differentiated generic skills from case- or time-specific skills. However, all of the models or guides above described a mix of skill sets as if they are from the same dimension. Logically, generic skills such as active listening, empathy, professionalism and patient-centered communication should be precursors to learning the structure and timing of communications for initial consultations. Generic skills are relevant to both initial consultations as well as more sporadic intra-operative communication. In other words, use of case- and time-specific guides that help students structure their communications with patients in a simplified manner should follow after they have some competency in the basic generic skills that apply to all forms of clinical communication. Generic skills often require the most time to learn, given their qualitative nature [8]. If learning generic skills were assumed to be a necessary precursor for the case- and time-specific structural guide of the DCCC, and thus were removed from the list in Figure 3, then the consultation guide would resemble the shortened list in Table 3 below. This would be a new streamlined, but effective consultation guide that assumes that generic skills are learned earlier. Assessment could be a tally of 15 items on a 0–4 scale (60 max.), similar to the OS-12 for students of allied health care.

In summary, clinical consultation guidance schemes for dental students need to fit with the assumptions for dental clinical situations. Based on analysis of the consultation guide literature, recommendations described above are (1) two phase learning in CST for dental students, with focus on learning patient-centered generic skills in the first phase and (2) an efficient and effective guidance and structural plan for the chronological order of longer consultations in dentistry in the second phase.

### 4.5. Optimal Curricular Structure for Clinical Communication Learning Effectiveness

#### Longitudinal vs. One-Time Learning

Longitudinal learning is almost taken for granted in educating dental students about caries/restorative dentistry, periodontology, prosthodontics and oral physiology. However, in dental educational programs for communication, there is limited progressive development during the curriculum in most cases [8,15]. The premise about superiority of a longitudinal communications curriculum versus one-time learning was also a conclusion drawn from the systematic review by Carey et al. [7]. Later Ayn et al., 2017 [15] and Khalifah and Celenza in 2019 [8] emphasized that dental students should start receiving communication training in the pre-clinical years, continuing into their clinical years and be evaluated during interactions with real patients in order to provide maximum competence.

A curricular example of teaching communication skills was developed by Ayn et al. [15] after they compiled their review from the dental education literature and other higher education experience. Inspired from the review, the suggested model curriculum reemphasized that knowledge learned during earlier, generally preclinical stages of dental education should be the foundations upon which both experiential and lecture-based learned strategies are based throughout students’ clinical learning experiences. A similarly structured timeline for communication skills in medical education described by Deveugele et al. [46] was shown to have the benefits of early detection and correction of communication skill issues and improved retention while providing a greater understanding of the importance for communication skills in the patient–professional relationship. Earliest possible experiences with the dental clinic should also improve motivation of young dental students for optimal communication with patients and personnel [47]. Ayn et al. [15] believe that this longitudinal learning approach should also be the mainstay of learning and becoming competent in communication skills in dental education.

Studies on German-speaking dental schools [16,48] have indicated that a longitudinal curricular approach in dental student communication skills is best in both teacher and student evaluations [16]. However, only 18 of the 34 German-speaking schools had implemented a fully or partially longitudinal curriculum, while the other sites only offered standalone courses as of 2016 [16]. Of the 34 dental schools, only six assessed communication skills in a summative way. Three of those schools also used formative assessments for their students.

It was apparent in the literature that there is a need to assess dental students’ skills in a longitudinal communication skills curricula. Although this review does not focus on assessment as a theme, the importance of competence assessment begs the question: “Did dental students learn what they needed to learn to improve their clinical communication skills?” 

**Figure 4:**
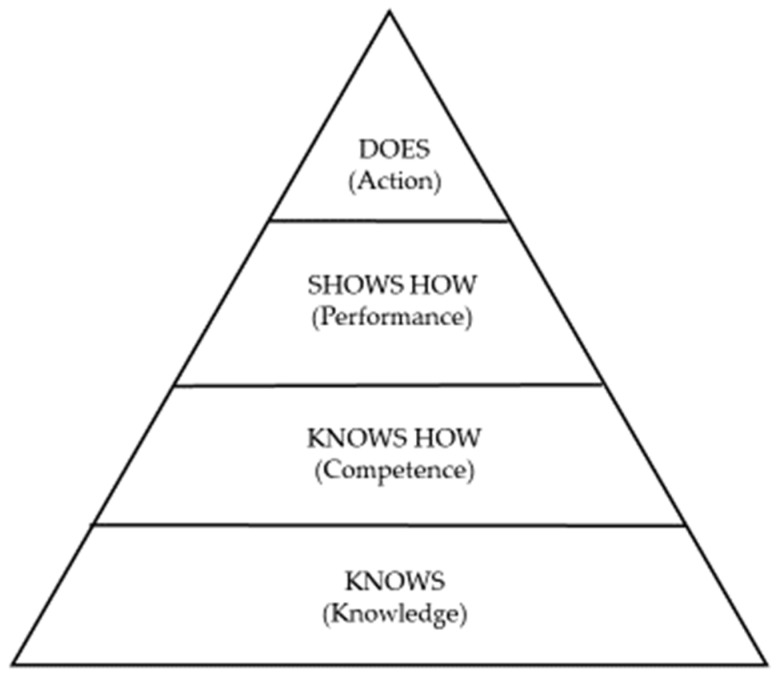
Miller’s pyramid model of clinical assessment [49].

Two of the tabled reviews [13,33] cited Miller [49] who proposed a pyramid framework for assessing clinical competence, which also applies to the learning of communication skills in dental education (see Figure 4). At the lowest level of the pyramid is knowledge (what a student knows), followed by competence (when a student knows how to use their knowledge), performance (when a student shows a teacher how they applied their knowledge) and action (when a student implements the practiced knowledge without tutelage). So, the ultimate test of clinical competence is that the knowledge is used in practice rather than what happens in artificial testing situations. Practical methods of assessment target this highest level of the pyramid. Miller [49] posited that other common methods of assessment, such as multiple choice questions, simulation tests and objective structured clinical examinations (OSCEs) target the lower levels of the pyramid. Thus, this pyramid model assumes that actual practice is a much better reflection of routine performance than assessments done under academic testing [13,33], which would also be the long-term goal of a longitudinal curricular program in CST.

## 5. Conclusions and Recommendations

The following conclusions and recommendations can be drawn from this study:

(1) The work environment for dentists is unique in that communication occurs most often while the patient is sitting passively in the dental chair, which for some, can be symbolic for vulnerability. Often the patient cannot talk since they are opening their mouths for dental treatment. It is important that regardless of whether there is an initial consultation or whether treatment needs to be interrupted for a consultation, the basis for patient communication and at dental teaching clinics should be a patient-centered approach. It should emphasize high-quality dialogue based on the generic skills of active listening, empathy and mirroring of patient perceptions.

(2) Differentiation of skill sets required in the dental clinical environment and appropriate adjustment to needs for generic skills, case-specific skills, time-specific skills and emerging or advanced skills would be best fine-tuned with the aid of video clip supervision both in dentist–patient role-play in pre-clinical years and with patients in clinical years.

(3) Motivational interviewing as a consultation guide is optimally useful in health promotion and appreciative inquiry approaches to oral health behavioral change interventions.

(4) The basic protocol for systematic initial anamnestic dental consultations should start with a modified Calgary–Cambridge–like Guide, such as the Dental Consultation Communication Checklist. However, learning in dental communication skills should not be limited to initial anamnestic consultations, which also requires rethinking consultations during the treatment phase much as the Four-plus-one Habit model suggests. A DCCC short form was proposed for learning initial consultation structure and timing in dental educational CST. The short form assumes prior learning and competence in generic skills such as active listening, empathy, rapport building and motivation.

(5) Longitudinal learning should be a strategic goal in communication skills education, in which learning with time and experience progress over the curriculum along with general clinical experience levels of students. Communication skills need to be taught and learned with the same rigor as other core dental skills.

## Figures and Tables

**Table 1:** Reviews and findings of communications skills learning for the four themes.

Theme	Review Source *n* = 12	Study Findings
**Special characteristics about the dental clinic relevant to learning communication skills**	Khalifah & Celenza, 2019 Carey et al., 2010	▪ Systematic reviews a decade apart reiterated the need for communication skills learning not just as in initial medical consultations, but also dental intra-operative communication due to differences in medical and dental contexts.
	Cheng et al., 2015	▪ Delineated medical and dental contexts and cited sociolinguists Coleman and Burton, who studied dentist–patient communication noting that there was very little patient participation unlike in medical clinics. Consultations in medicine and dentistry also differ in that hands-on treatment is always expected in dentistry, making the demand on communication skills not only applicable in initial consultation, but also during operative phases.
**Essential components of dental student learning communication skills**	Khalifah & Celenza, 2019	▪ Systematically identified 26 communication skills that fell under four categories: generic skills, case-specific skills, time-specific skills and emerging skills (see Figure 1). Tabled each of the 50 relevant studies for type of communications skills taught, teaching method (e.g., role-play, video supervision, lectures), assessment method and outcomes. Active listening, empathy and professionalism were prominent, indicating a trend toward patient-centered communication.
Patient-centered care (PCC)–active listening and empathy	Scambler et al., 2016	▪ Systematic review concluded that PCC is about delivering humane care involving good communication and shared decision making. Noted there was neither work assessing these concepts empirically nor were they clearly understood in dental settings. Presented a model of four levels of information and choice provision and/or agreement between clinician and patient.
	Mills et al., 2014	▪ Systematic review revealed a lack of understanding of PCC particularly in general dental practice. Reported that current patient outcome measures as indicators of patient-centeredness are inadequate. Special dentistry qualitative research about treatment of phobic or economically disadvantaged patients provided some evidence of good outcomes using PCC for vulnerable patients.
	King & Hoppe et al., 2013	▪ A narrative review of the medical literature showed considerable evidence to support positive associations between PCC physician communication and positive outcomes with patients, such as improved recall, understanding, satisfaction and compliance.
Other aspects of education of dental students’ communication skills	Khalifah & Celenza, 2019	▪ Systematic review showed dental students were positive about actively learning communication skills, regardless of using role-play or clinical video supervision However, video supervision of actual patient–dentist interactions and especially one to three concentrated course days, were best for learning optimal communication skills.
	Carey et al., 2010	▪ Systematic review indicated that it was best that skills be evaluated during interactions with real patients, thus calling for at least some clinical coursework after initial role-play in earlier coursework.
Role of clinical instructors	Burkert, 2021	▪ Narrative review reported that ultimate learning of optimal communication skills requires teachers to be role models, effective supervisors, powerful tutors, and supportive persons who use diverse teaching methods with an individual approach to educating their students.
	Ayn et al., 2017	▪ Clinical instructors present communication role models with very little institutional control over learning quality. Teacher education required in order to maximize student learning.
**Clinical consultation guides used in communication skills learning**	Buduneli, 2020 Kalifah & Celenza, 2019	▪ Reviews identifying literature relevant to learning consultation styles, guides and models that have been used to try to organize learning of CST: motivational interviewing (MI), Calgary–Cambridge Guide (C-CG), Macy Foundation model, Manitoba model, Dental Consultation Communications Checklist (DCCC) and Four-plus-one Habit model (4 + 1HD)
	Gillam & Yusuf, 2019	▪ MI developed into a patient-centered communication approach for patients, such as alcoholics and smokers, who wanted to change their behavior. Promotes the use of the six-functions model as well as a strategy with the acronym “OARS”, i.e., asking Open-ended questions, providing Affirmations about patient goals, use of Reflective listening including “change talk” and discussion of Summaries that capture the process in reflection.
	Gao et al., 2014	▪ MI outperformed conventional education in improving at least one outcome in: four studies on preventing early childhood caries, a study on adherence to dental appointments, and two studies on prevention of facial injury after abstinence from illicit drugs and alcohol abuse. MI had a superior effect on oral hygiene in five CBT trials out of seven.
	King & Hoppe, 2013	▪ The six-functions model as the foundation of all consultation models in which goals for medical encounters are 1) fostering the relationship, 2) gathering information, 3) providing information, 4) making decisions, 5) responding to emotions, and 6) enabling disease and treatment related behavior (help to self-management).
**Optimal curricular structure for effective learning of clinical communication skills**	Khalifah & Celenza, 2019	▪ Systematic review of curricula; proposing that learning of CST is best in a longitudinal curriculum
	Rütterman et al., 2017	▪ Systematic review of German-speaking schools found that 30% surveyed pursued a longitudinal curriculum, i.e., multiple points over time in which CST was taught.
	Ayn et al., 2017	▪ Systematic scoping review suggested that CST may be most effective if integrated throughout the curriculum suggesting effectiveness may be optimized as students gain clinical experience as in other clinical disciplines. Students seemed to benefit from increased self-evaluation and reflection regarding their CST performance. Assessments that are similar in nature to those of other clinical competencies might improve perceived importance of CST.

**Table 2:** Observation Scheme 12 adapted from Iversen et al., 2020 [40] with corresponding C–CG domain’s micro-skills are designated in a codebook for each item.

Skill Level Representing Multiple Micro-Skills	Corresponding C–CG Domains
(1) Identifies problems the patient wishes to address.	Initiating the session
(2) Clarifies patient’s prior knowledge and desire for information.	Gathering information
(3) Uses easily understood language, avoids jargon.	Gathering information
(4) Uses appropriate (supportive) non-verbal behavior.	Building a relationship
(5) Provides support: expresses concern and willingness to help.	Building a relationship
(6) Structures the interview in logical sequence.	Providing structure
(7) Attends to passage of time and keeps the interview on track.	Providing structure
(8) Shares thoughts and reflections with the patient.	Explanation and planning
(9) Checks patient’s understanding.	Explanation and planning
(10) Negotiates a mutual plan of action.	Explanation and planning
(11) Contracts with patient about the next steps.	Closing the session
(12) Summarizes session briefly and clarifies plan with patient.	Closing the session

**Table 3:** **A** Select Dental Consultation Communications Checklist as a case- and time-specific structural guide that does not include generic skills, such as active listening and other PCC skills.

Action:	Corresponding Domains:
(1) Greet the patient.	Investing in relationship
(2) Introduce yourself and that you are prepared to listen.	Investing in relationship
(3) Ask patient to explain reason for visit from own perspective.	Investing in relationship
(4) Explain what will happen during the visit; no jargon.	Investing in relationship
(5) Be ready to reformulate questions with patient confusion.	Gathering information
(6) Handle personal questions sensitively: What has patient heard?	Gathering information
(7) Explain what you want to do and why before you do it.	Examination/Explanation/Planning
(8) Check patient comfort before examination.	Examination/Explanation/Planning
(9) Explain findings without technical language.	Examination/Explanation/Planning
(10) Reassure patient if necessary and use X-rays, other aids.	Examination/Explanation/Planning
(11) Negotiate a mutual plan of action and next steps.	Examination/Explanation/Planning
(12) Check patient understanding.	Examination/Explanation/Planning
(13) Point out that conversation is coming to an end.	Investing in closure
(14) Summarize session briefly; invite further questions/concerns.	Investing in closure
(15) Explain what will happen next; make new appointment.	Investing in closure

## Data Availability

Not applicable.
